# The Legal Liability of the Anæsthetist

**Published:** 1908-03-28

**Authors:** 


### THE LEGAL LIABILITY OF THE ANÆSTHETIST.

AT the adjourned meeting of the Medico-Legal Society on March 24, the discussion of Dr. L. Freyberger's paper on "Deaths during Anæsthesia" was resumed and was directed somewhat more into legal channels than at the first meeting. Mr. R. H. Wellington opened the debate with a carefully thought out analysis of these legal aspects of the subject. Reminding the meeting of Dr. F. W. Hewitt's speech after the original paper, in which the opinion was advanced that the majority of deaths from or under an anæsthetic are the fault neither of the operator nor of the patient, but of the anæsthetist, the Deputy Coroner for Westminster and the South-West District explained that legally anyone may give any anæsthetic. All that the Medical Acts provide is that the public shall have the means of finding out whether any given individual is or is not regarded by the General Medical Council as qualified to practise; if, in spite of this, the public chooses to employ an unqualified person, it must take the consequences. In a word, there are penalties for an unqualified man who pretends to be qualified, but none for open unqualified practice. Mr. Wellington then dwelt on the great difficulty there must always be in establishing want of reasonable skill in any individual case, and of excluding contributory causes such as the idiosyncrasy or the morbid condition of the patient, and the effect of the surgical proceedings. He next suggested that the Privy Council should make it penal for any unqualified man to administer any anæsthetic, whether local or general, and that the County Councils might well appoint a Registrar, who should be a medical man with a legal training, to abstract and report on the masses of waste material which exist in the depositions taken at inquests.

The regulations of the General Medical Council on the subject of "covering" were explained; it is just as unethical for a qualified man to give an anæsthetic for an unqualified surgeon or dentist, as for a qualified operator to employ an unqualified anæsthetist. Similarly an anæsthetist administering for the purpose of an illegal operation is not liable if he does not know its nature, but if he does know the operation to be illegal he is equally guilty with the surgeon of felony if the patient survives and of murder if she dies. It is very doubtful whether a dentist having the L.D.S. without a full qualification as physician and surgeon is qualified to give any anæsthetic at all; whether he may give nitrous-oxide; or whether he may give any general anæsthetic he may select.

If a patient is the subject of a pathological condition, and is warned that there is unusual risk for that reason, by consenting to operation he exonerates both surgeon and anæsthetist in case of a fatal result. If a pathological condition contributes to such an issue, and was previously unsuspected, death is due to misadventure. If during an operation the anæsthetist warns the surgeon of impending danger, the *onus probandi* is perhaps thrown upon the latter if he proceeds; but as both operator and anæsthetist must work together and do their best for the patient, no action can be sustained for *mala praxis* if the surgeon decides that a continuation of the operation is in the best interests of the patient. If in a hospital an unqualified pupil is administering an anæsthetic under the supervision of a qualified anæsthetist, the former is not acting illegally and the latter is not "covering." There is a risk that busy surgeons anxious to get away to their private practice may unduly hurry young anæsthetists in hospitals, and so increase the risk of induction. Before an anæsthetist can be made answerable to the law for damages or punishment, it must first be proved beyond reasonable doubt that death is attributable to the anæsthetic as a poison, *per se*, and then that the administrator, whether qualified or unqualified, did not use reasonable care, skill, and attention in exhibiting it. Administration by unqualified persons is carried on to a very large extent throughout the Kingdom, and should be made a penal offence.

Dr. Hewitt, by permission of the President, then spoke for the second time during the debate, for the purpose of submitting a resolution. He commented on the unsatisfactory state of the law as set forth in Mr. Wellington's paper and the measures necessary in his judgment to remedy the present state of affairs. Anyone may now administer an anæsthetic, whether qualified or unqualified, experiencd or inexperienced, reputable or disreputable: whereas, on the other hand, it is thought necessary to protect the public in matters of food and drug adulteration. Although the General Medical Council advises, it does not compel the instruction of students in anæsthetics. Out of the twenty-two examining bodies in the United Kingdom, only eight do insist on such instruction: in England seven out of eleven require it, in Scotland one out of six, and in Ireland none out of five. Apart altogether from the question of specialism in this branch of practice, the average practitioner leaves his hospital not so well equipped for the administration of anæsthetics as he might be. Accordingly he moved that the two following resolutions be forwarded, in the name of the Medico-Legal Society, to the Privy Council and to the General Medical Council respectively.

"That, as the administration of any drug or drugs with the object of producing generalised insensibility to pain, during a surgical operation or during childbirth, cannot be safely undertaken without medical knowledge and skill, your petitioners humbly pray that legislation be granted whereby the administration of any drug or drugs with either of the aforesaid objects by any person other than a duly registered medical practitioner or someone acting immediately under his supervision, direction, and instruction, be made a penal offence."

"That, in view of the frequency with which deaths continue to take place during general anæsthesia, this Society is of opinion that it is highly desirable, in the public interest, that every member of the medical profession, before he is registered, shall have received thorough instruction in the administration of anæsthetics. They therefore earnestly beg the General Medical Council to consider whether they can see their way to include a course of instruction in anæsthetics amongst their 'Requirements in regard to Professional Education.'"

This motion having been seconded by Mr. Wellington, Sir A. D. Fripp rose to give his heartiest support to the proposals for the compulsory training of medical students. He thought the first half also of the motion should have the support of the whole profession. At the same time, it must be recognised that such legislation would deprive many members of the public of the chance of getting a tooth extracted under gas for the modest sum of half-a-crown. The medico-political aspects of the question are of considerable importance: possibilities are opened up of greater strain upon hospitals and other charities if cheap anæsthesia cannot be procured. The importance of the anæsthetist has increased steadily with that of the surgeon. Sir Alfred far prefers the levelling up of the standard of education in respect of anæsthesia throughout the profession to the restriction of this branch of practice to specialists alone.

Dr. Probyn Williams, while approving of the motion, was unable to agree with many of Dr. Hewitt's arguments. He would not admit the statement that the result of anæthesia depends on the anæsthetist only, and not on the drug, the operator, or the patient. He also dwelt upon the efforts of the Society of Anæsthetists, which resulted in the present regulations of the Royal College of Surgeons.

Dr. F. J. Smith thought the first half of the motion excellent as a pious opinion, but regarded the idea of the legislature interfering with the quackery rampant in the land as chimerical. He protested very strongly against the usage of many coroners in holding inquests on every case of death during anæsthesia. It is not done in Scotland, and it is only done in England for purely personal reasons. A preliminary private inquiry is within their discretion, and in the great majority of cases is all that is required. The law as it at present stands requires, and rightly requires, that a qualified medical man shall do his best for his patients; that he shall use that skill that he possesses, and no more is expected. Thus from Dr. Hewitt the law expects more skill and proficiency in the administration of anæsthetics than from a country practitioner.

Professor Glaister (Glasgow) was sure that Dr. Hewitt is in error as to the number of examining boards in Scotland. He also dissented very strongly from the assertion of Dr. Hewitt at the previous meeting that nine deaths out of ten during anæsthesia are preventable, and, in other words, the fault of the anæsthetist.

Dr. F. J. Waldo (Coroner for the City and for Southwark) said that in 1903 the Society of Coroners advised that an inquest should be held in every case of death during anæsthesia. He disagreed with Dr. Smith as to the Coroner's powers, and believes that the provisions of the Coroners' Act of 1887 make inquests in these cases imperative.

Professor Harvey Littlejohn confirmed the statement that in Scotland most inquiries of this kind take place privately, and approves of this. The Procurator Fiscal takes the advice of some medical jurist of standing on each case, and need not order a public inquiry unless he thinks one necessary. He is strongly of opinion that the good attained by universal public inquiries is outweighed by the evil results of terrifying the public and instilling into them mistaken ideas as to the risks of anæsthesia and the competence of administrators.

Drs. Blumfeld, Templeman, and Silk having contributed to the debate, the motion was carried.

